# At-TAX: a whole genome tiling array resource for developmental expression analysis and transcript identification in *Arabidopsis thaliana*

**DOI:** 10.1186/gb-2008-9-7-r112

**Published:** 2008-07-09

**Authors:** Sascha Laubinger, Georg Zeller, Stefan R Henz, Timo Sachsenberg, Christian K Widmer, Naïra Naouar, Marnik Vuylsteke, Bernhard Schölkopf, Gunnar Rätsch, Detlef Weigel

**Affiliations:** 1 Department of Molecular Biology, Max Planck Institute for Developmental Biology, Spemannstr. 37-39, 72076 Tübingen, Germany; 2 Friedrich Miescher Laboratory of the Max Planck Society, Spemannstr. 39, 72076 Tübingen, Germany; 3 Department of Plant Systems Biology, VIB, Technologiepark 927, 9052 Ghent, Belgium; 4 Department of Molecular Genetics, Ghent University, Technologiepark 927, 9052 Ghent, Belgium; 5 Department of Empirical Inference, Max Planck Institute for Biological Cybernetics, Spemannstr. 38, 72076 Tübingen, Germany

## Abstract

Gene expression maps for model organisms, including *Arabidopsis thaliana*, have typically been created using gene-centric expression arrays. Here, we describe a comprehensive expression atlas, *Arabidopsis thaliana *Tiling Array Express (At-TAX), which is based on whole-genome tiling arrays. We demonstrate that tiling arrays are accurate tools for gene expression analysis and identified more than 1,000 unannotated transcribed regions. Visualizations of gene expression estimates, transcribed regions, and tiling probe measurements are accessible online at the At-TAX homepage.

## Abstract 2

A developmental expression atlas, At-TAX, based on whole-genome tiling arrays, is presented along with associated analysis methods.

## Background

The generation of genome-wide gene expression data for the reference plant *Arabidopsis thaliana *yielded important insights into transcriptional control of development, with genome-wide expression maps having become an indispensable tool for the research community. Specific gene expression profiles for various plant organs, developmental stages, growth conditions, treatments, mutants, or even single cell types are available (for example [1-7]). These data have helped to elucidate transcriptional networks and attending promoter motifs, to uncover gene functions, and to reveal molecular explanations for mutant phenotypes (for review [8]).

The most widely used platform for *Arabidopsis *is the Affymetrix ATH1 array [9,10]. Its design used prior information in the form of experimentally confirmed transcripts and gene predictions, and was intended to provide information on most known transcripts. Although the ATH1 array includes more than 22,500 probe sets, it lacks almost one-third of the 32,041 genes found in the most recent TAIR7 annotation [11]. All users of ATH1 arrays are confronted with a problem; as the number of newly discovered genes is rising, expression analysis becomes more and more restricted.

More unbiased detection of transcriptional activity can be achieved by sequencing techniques such as massively parallel signature sequencing and serial analysis of gene expression or, alternatively, by microarrays that interrogate the entire genomic sequence, so called 'whole genome tiling arrays' [12-14]. In contrast to arrays that are focused on gene expression, which contain only probes complementary to annotated genes, whole-genome tiling arrays are designed irrespectively of gene annotations and contain probes that are regularly spaced throughout the nonrepetitive portion of the genome [15]. This includes intergenic and intronic regions, and whole-genome tiling arrays can therefore measure transcription from annotated genes, identify new splice and transcript variants of known genes, and even lead to the discovery of entirely new transcripts.

Outside the context of plants, tiling arrays have been used to detect transcriptional activity in the genome of several organisms, including baker's yeast, *Caenorhabtidis elegans*, *Drosophila melanogaster*, and humans [16-22]. Apart from the discovery of new transcripts, tiling arrays are useful for mapping the 5' and 3' ends of transcripts, and for the identification of introns (for example [23]). Perhaps most importantly, these studies have expanded our understanding of genome organization. Apparently, genomes give rise to many more transcripts than was previously assumed. Most of these are noncoding RNAs emerging from intergenic regions, a large portion of which had previously been underrated as 'junk' DNA [24]. Although the functional relevance of the majority of these transcripts remains unclear, their abundance and the fact that they have escaped *ab initio *gene predictions highlight the advantages of whole-genome tiling arrays. Another group of transcripts that has frequently been ignored in the past are nonpolyadenylated transcripts. Up to 50% of distinct transcripts in human and *C. elegans *lack polyA tails; this phenomenon is neglected by most gene expression studies, which typically use polyA(+) RNA as starting material or oligo-dT-primers for reverse transcription [19,20].

The first tiling array analyses of *Arabidopsis *and rice combined with sequencing of full-length cDNAs delivered important information about gene content, gene structure, and genome organization [14,25-30]. Furthermore, gene expression profiling with tiling arrays of *Arabidopsis *mutants led to the identification of hundreds of noncoding transcripts that are normally silenced or removed by the exosome [31,32].

In line with findings in yeast and animals, Yamada and colleagues [14] reported that many *Arabidopsis *genes are also transcribed in anti-sense orientation, implicating anti-sense transcription in gene regulation. More recent studies in yeast and mammals suggested that at least some of the signals may be due to artifacts of reverse transcription methods used to generate the probes for array hybridization [33,34].

Here, we use the Affymetrix GeneChip^® ^Tiling 1.0R Array (Affymetrix Inc., Santa Clara, CA, USA) to provide an initial whole-genome expression atlas for *A. thaliana*, dubbed '*Arabidopsis thaliana *Tiling Array Express' (At-TAX), using RNA samples from 11 different tissues collected at various stages of plant development. We directly compare the performance of the tiling array, which contains one 25-base probe in each nonrepetitive 35 base pair (bp) window of the reference genome, with that of the 'gold standard' ATH1 array. We also report on the expression profile of over 9,000 annotated genes that are not represented on the ATH1 array. Applying a recently developed computational method for transcript identification to the tiling array data allowed us to identify regions not previously annotated as transcribed [35]. Our data also suggest that most *Arabidopsis *transcripts expressed at detectable levels are polyadenylated. To benefit the *Arabidopsis *research community, we provide an online tool for visualization of gene expression estimates, along with a customized genome browser [36].

## Results

### A tiling array based expression atlas of polyadenylated transcripts

We isolated RNA from ten tissues and different developmental stages, ranging from young seedlings to senescing leaves, and roots to fruits of the *A. thaliana *Col-0 referenced strain. In addition, we made use of inflorescence apices from the *clavata3 *(*clv3*) mutant [37] to enrich for shoot and floral meristems (Additional data file 1). We used both GeneChip^® ^Tiling 1.0R and ATH1 gene expression arrays to obtain triplicate expression estimates from all samples. Because our priority was to detect transcribed regions, we decided to use double-stranded DNA (dsDNA) as hybridization targets for the tiling arrays. Consequently, we did not obtain information about the strand from which a signal originates. However, several recent reports have raised the question of how reliable the detection of antisense transcripts on tiling arrays is [33,34]. Another advantage is that DNA targets exhibit higher specificity than RNA targets [38].

To profile the expression of annotated genes on tiling arrays, we extracted probe information for all genes that can be analyzed in a robust manner (see Materials and methods [below] for details). Consequently, we ignored small transcription units such as tRNA genes, which are represented by an insufficient number of probes. Having each gene represented by a set of probes allowed us to apply a standard algorithm, robust multichip analysis (RMA), to both microarray platforms, thereby minimizing differences resulting from different analytical procedures [39]. A total of 20,583 genes were represented on both platforms; an additional 136 and 9,645 genes were exclusively represented on ATH1 and the tiling array, respectively. Resulting RMA log2 expression values for tiling and ATH1 arrays spanned 11 to 12 log2 units in both cases.

**Figure 1:**
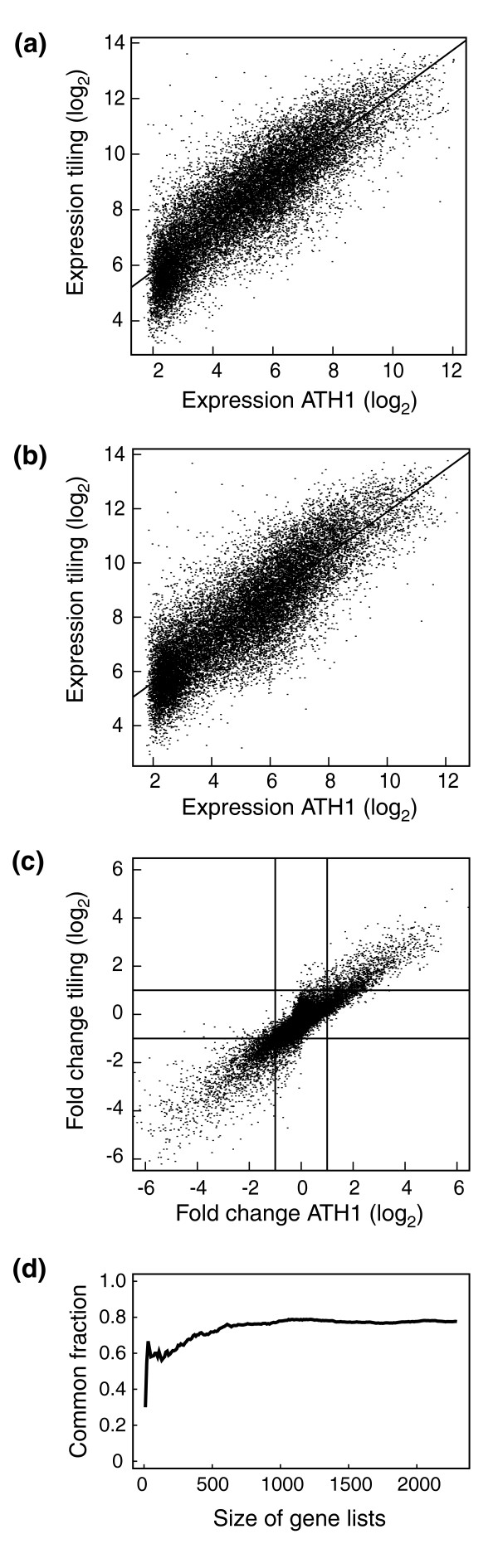
Comparison of expression estimates on tiling and ATH1 array platforms. Scatter plot of expression estimates in **(a) **roots and **(b) **inflorescences. **(c) **Correlation between expression changes between roots and inflorescences. **(d) **CAT (correspondence at the top) plot for genes identified differentially expressed in roots and inflorescences. Proportion of genes in common is shown as a function of increasing size of subsets containing the *n *genes with the highest *P *values.

To compare the expression values derived from ATH1 array and tiling array, we generated scatter plots and calculated pair-wise Pearson correlation coefficients (PCCs) for all samples (Figure 1a,b and Table 1). Expression values for all genes in a given sample were well correlated across platforms, with PCCs ranging from 0.854 to 0.882 (*P *< 10^-15^), indicating that both produce comparable results. Transcripts with expression estimates close to background correlate the least between platforms, as a result of higher variance of tiling array estimates (Figure 1a,b).

**Table 1:** Correlation of ATH1 and tiling arrays expression values across the analyzed samples

Sample	Description	PCC	Potential tissue-specific transcripts
1	Roots	0.86	378
2	Seedlings	0.88	5
3	Expanding leaves	0.87	13
4	Senescing leaves	0.87	301
5	Stem	0.87	34
6	Vegetative shoot meristem	0.86	19
7	Inflorescence shoot meristem	0.87	14
8	Whole inflorescences	0.85	152
9	Whole inflorescences (*clv3-7*)	0.86	
10	Flowers	0.88	51
11	Fruits	0.86	98

Presented are the correlations for gene expression estimates between ATH1 and tiling array platform, and number of candidates for tissue-specific genes (z score > 2.5 across all samples and most abundant in this tissue) detected in each sample. PCC, Pearson correlation coefficient.

We were particularly interested in the power of the tiling array to detect differential gene expression. To this end, we compared two samples, roots and inflorescences, which are known to have very different expression profiles [5]. Applying the RankProduct method (RankProd) [40,41], we detected 2,484 and 2,294 differentially expressed genes (*P *< 0.05) on ATH1 and tiling arrays, respectively, with 1,780 genes in common. A PCC of 0.92 (*P *< 10^-15^) indicated a good agreement for detecting expression differences of individual genes across platforms (Figure 1c). In addition, we generated a 'correspondence at the top' (CAT) plot using *P *values to rank the genes (Figure 1d) [42]. In the top 200 and 1,500 lists, 150 and 1,308 genes, respectively, were found in common, further supporting high concordance between the two types of arrays.

**Figure 2:**
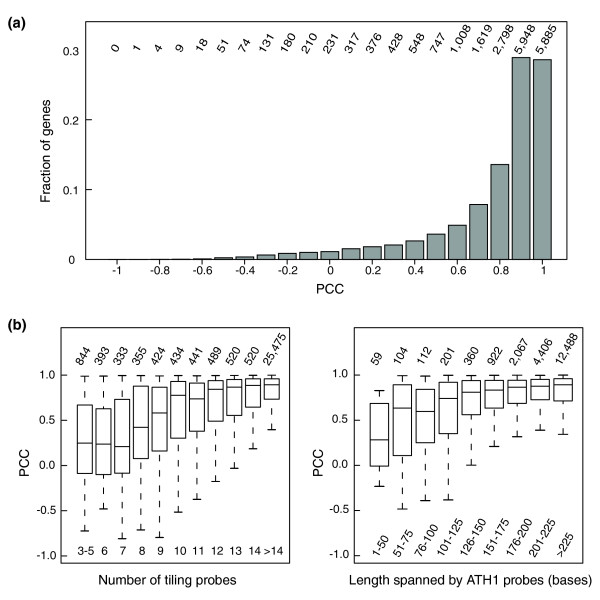
Platform concordance and factors affecting it for genes represented on both ATH1 and tiling arrays. **(a) **Pearson correlation coefficients (PCCs) of expression estimates. **(b) **Box plots showing expression correlation for genes that were either categorized by the number of probes on tiling arrays or categorized by the total length of nonredundant sequence spanned by ATH1 probes. The boxes have lines at the lower quartile, median, and upper quartile values. Whiskers extend to the most extreme value within 1.5 times the interquartile range from the ends of the corresponding box. Box plots are based on genes represented on both the ATH1 and the tiling array, with the total number of genes per category on the respective platform indicated at the top.

Comparing the platforms across all samples, we found that more than 70% of all genes showed a correlation of 0.8 or greater (Figure 2a). Genes with low correlation between platforms tend to be those that are represented by a comparably small number of tiling probes (Figure 2b). Qualitatively, the same is true for genes that, because of the improved annotation, are represented by only a limited number of probes on the ATH1 array (Additional data file 4) or by strongly overlapping probes on ATH1 (Figure 2b). These results indicate that gene expression estimates based on ten or more tiling array probes are highly robust. More than 27,000 annotated genes fulfill this requirement for the Affymetrix Arabidopsis 1.0R tiling array, making it a powerful tool for gene expression studies.

### Expression of annotated genes not represented on the ATH1 array

**Figure 3:**
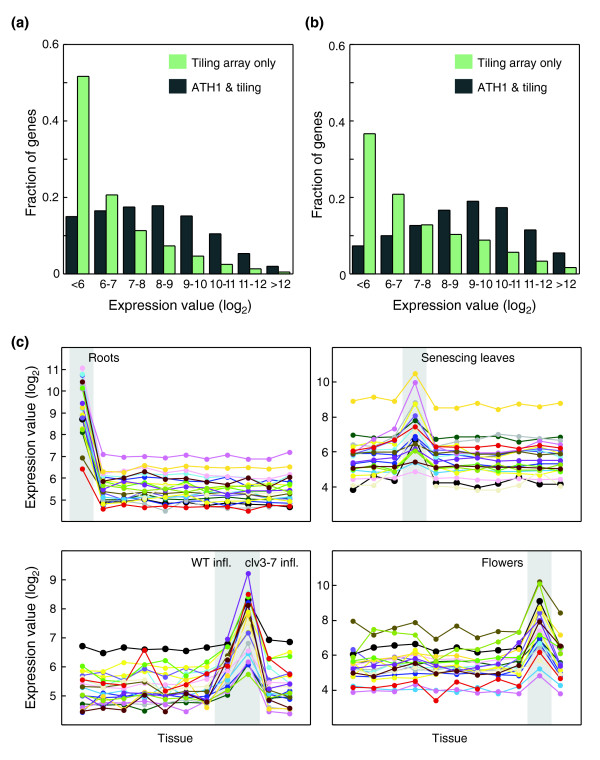
Analysis of genes represented only on tiling arrays. **(a) **Average or **(b) **maximum expression levels for all genes across all samples. **(c) **Expression values of genes with an apparent tissue-specific or stage-specific expression pattern across all samples. Twenty genes with the highest z scores and maximum expression in root, senescing leaf, inflorescence, or flowers are shown.

The tiling array allows the analysis of 9,645 genes, corresponding to 31.9% of all annotated genes, that are not represented on the ATH1 array. The average expression levels of these genes across all 11 samples are clearly lower than of those that are also present on the ATH1 array. Although only 15% of genes represented on both the tiling and ATH1 array platform have average expression level of less than six log_2 _units, this applies to more than 50% of the genes found only on the tiling array (Figure 3a). This is consistent with priority during the ATH1 design being given to genes with prior expression evidence [9]. Nevertheless, many genes absent from ATH1 are expressed more highly in at least one sample (Figure 3b).

Of the 9,645 genes, 1,065 genes had z scores exceeding 2.5 across the 11 samples, making them good candidates for having tissue-specific or stage-specific expression patterns (Additional data file 9, Table 1, and Figure 3c). The number of easily detectable transcripts was higher in roots or senescing leaves than in young leaves or seedlings, which is in agreement with previous observations [5].

### Identification of new transcripts across different developmental stages

To identify transcripts that are not present in the current genome annotation, we adopted a computational method, margin-based segmentation of tiling array data (mSTAD), for the segmentation of tiling array data into exonic, intronic, and intergenic regions [35]. Extending a segmentation method developed for yeast tiling arrays [43], we modeled spliced transcripts with ten discrete expression levels and incorporated a more flexible error model. Moreover, mSTAD is a supervised machine-learning algorithm with internal parameters that are estimated on hybridization data together with information on the location of annotated genes. After training, it can make predictions based on hybridization data alone.

**Figure 4:**
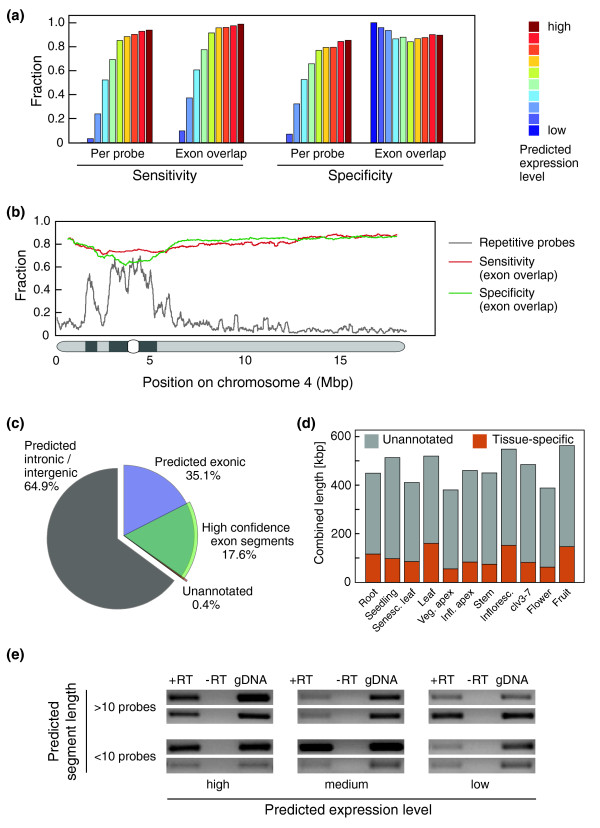
*De novo *segmentation of tiling array data. **(a) **Segmentation accuracy for roots across ten discrete expression levels (see inset). Sensitivity is defined as the proportion of exonic probes contained in predicted segments relative to all annotated exonic probes, or the proportion of identified exon segments to all annotated exons. Specificity indicates how many predicted expressed probes or predicted exons are annotated as such. **(b) **Sensitivity and specificity of predicted exon segments for roots in comparison with annotated exons, plotted in a sliding window across 2,000 exons along chromosome 4 together with information on repetitive probes (window of 5,000 probes; see inset). The heterochromatic knob, the centromere and peri-centromeres are depicted below the x-axis (for other chromosomes, see Additional data file 5). **(c) **Proportion of predicted exon segments, high-confidence exon segments (see text for definition), and unannotated exon segments (high-confidence predictions that do not overlap with any annotated exon by at least 25 base pairs). Numbers are based on combined length of each class. **(d) **Proportion of sample-specific exon segments among all unannotated high-confidence predictions. **(e) **Examples of RT-PCR validation of predicted novel transcripts.

When comparing a genome-wide sample of all mSTAD exon predictions with annotated genes, we found that the predictions were generally accurate for the more highly expressed half of genes (Figure 4a; see Materials and methods [below] for details). For each sample, we further analyzed a set of high-confidence exon predictions (Figure 4b and Additional data file 5). These contained a minimum number of four probes, had predicted discrete expression level between 6 and 10, and had at most 25% repetitive probes. From these high-confidence exon predictions, which make up 37% to 50% of the total length of all predictions depending on the tissue analyzed, more than 97% overlap at least 25 bp with annotated exons (Figure 4c). Between 26% and 36% of the remainder overlap with cDNAs and expressed sequence tags (ESTs) but not with annotated transcripts.

In summary there are between 1,107 and 1,947 predicted high-confidence exons per sample, for a total length of 242 to 406 kilobases (kb), that are neither included in the current annotation nor covered by sequenced cDNA clones. A complete list of all high-confidence exons with chromosome start and end position can be downloaded from the At-TAX homepage [36]. Among the unannotated high-confidence predictions, 14% to 31% are specifically detected in a single sample, with inflorescences and senescing leaves showing the highest proportion (Figure 4d). Whether these predictions indeed correspond to expressed transcripts was tested for some of these by RT-PCR. From high-confidence predictions that do not overlap with known cDNAs or ESTs, a subset of 47 segments was selected so that different lengths as well as different predicted expression strengths were covered. We could confirm by RT-PCR that more than three-quarters (37) of these 47 predicted segments as transcribed (Figure 4e and Additional data file 6).

### Analysis of nonpolyadenylated transcripts

**Figure 5:**
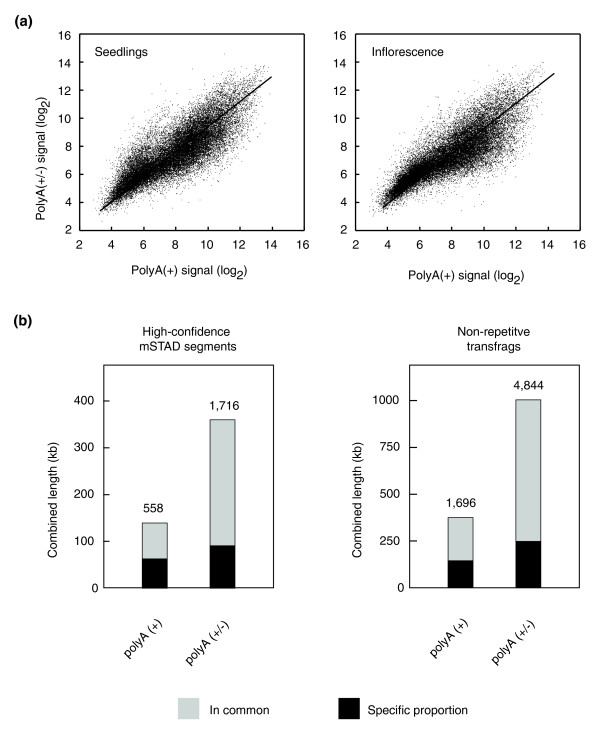
Non-polyadenylated transcripts. **(a) **Correlation between expression levels for polyA(+) and polyA(±) samples. **(b) **Proportion of unannotated transcripts found in common or exclusively in either polyA(+) samples and polyA(±) samples, respectively, as determined with two independent methods.

Previous analyses with whole-genome tiling arrays have focused on the polyadenylated portion of the *Arabidopsis *transcriptome [14,30-32]. However, studies conducted in several other organisms have suggested that there is a large fraction of nonpolyadenylated RNAs (for example [19,20]). In order to revisit this question in *Arabidopsis*, we isolated total RNA from two different tissues, whole seedlings and inflorescences, and depleted it for rRNA using a mix of locked nucleic acid (LNA) oligonucleotides. This RNA preparation was used for reverse transcription with either an oligo-dT primer (which targets only polyA [+] RNA) or random primers (which target both polyA [+]and polyA [-] RNAs). After conversion to dsDNA, samples were hybridized to tiling arrays. For both tissues analyzed, there was a good correlation between polyA(+) samples and polyA(±)samples (PCC = 0.84; *P *< 10^-15^; Figure 5a). Nevertheless, we found many transcripts that were more easily detected in polyA(+) samples than in polyA(±) samples. This probably reflects the fact that mean signal intensities are for unknown reasons generally lower toward the 3' end after random priming (Additional data file 7). Hence, expression values of short transcripts in particular may be underestimated with random-primed hybridization targets.

Only a small proportion of annotated genes produced a much higher polyA(±) signal compared with the polyA(+) fraction (Table 2). Large differences were detected for two structural RNAs: a U12 small nuclear RNA and an H/ACA-box small nucleolar RNA (Table 2). The majority of snRNAs undergo 3' end processing that is very distinct from polyadenylation [44,45], indicating that our method appears suitable for detecting nonpolyadenylated transcripts. Most other transcripts that were much more abundant in polyA(±) than in polyA(+) samples emanate from transposons and pseudogenes (Table 2). These results suggest that in *Arabidopsis *the overwhelming majority of known protein coding transcripts possess a polyA tail.

**Table 2:** Transcripts that are more abundant in polyA(±) samples than in polyA(+) samples

Locus	TAIR7 annotation	PolyA(+) (log2)	PolyA(±) (log2)
AT1G12013	H/ACA-box snoRNA	9.07	13.51
AT1G15405	Unknown gene	11.07	14.59
AT1G31960	Unknown protein	5.34	8.74
AT1G33860	Unknown protein	8.10	11.78
AT1G34700	Mutator-like transposase family	4.69	8.14
AT1G35080	Similar to unknown protein	3.70	7.03
AT1G35640	Unknown protein	5.91	9.29
AT1G41726	Pseudogene	6.73	10.30
AT1G61275	U12 snRNA	7.11	12.45
AT2G01022	Gypsy-like retrotransposon family	5.72	9.43
AT2G05567	Pseudogene	4.62	8.59
AT2G06250	Pseudogene	6.45	9.87
AT2G06370	Pseudogene	6.36	9.71
AT2G07709	Pseudogene	7.40	11.28
AT2G07711	Pseudogene	7.05	10.42
AT2G07712	Pseudogene	6.90	10.87
AT2G07717	Pseudogene	7.72	11.22
AT2G08986	Similar to unknown protein	6.64	10.15
AT2G10285	Similar to unknown protein	6.16	9.85
AT2G10720	Pseudogene	7.15	10.67
AT2G10790	Pseudogene	7.03	10.86
AT2G12240	CACTA-like transposase family	5.30	9.98
AT2G12320	Similar to unknown protein	6.56	10.05
AT2G12750	Gypsy-like retrotransposon family	7.20	10.71
AT2G13860	Gypsy-like retrotransposon	6.88	10.29
AT2G25255	Encodes a defensin-like (DEFL) family protein	5.65	9.04
AT3G24370	Similar to unknown protein	5.06	9.58
AT3G29570	Similar to ATEXT3	5.41	9.60
AT3G30846	Gypsy-like retrotransposon family	6.78	10.21
AT3G32010	Gypsy-like retrotransposon family (Athila)	5.37	9.41
AT3G32880	Gypsy-like retrotransposon family (Athila)	6.37	10.60
AT3G42251	Pseudogene	5.82	9.24
AT3G42750	Similar to unknown protein	4.44	7.85
AT3G43154	Pseudogene	5.21	9.22
AT3G43160	MEE38	7.42	11.95
AT3G43862	Athila retroelement ORF2-related	6.07	10.44
AT4G05290	Similar to unknown protein	5.39	10.08
AT4G06531	Pseudogene	4.21	7.93
AT4G06573	Athila retroelement ORF1 protein	7.25	11.01
AT4G06710	Pseudogene	6.53	11.72
AT4G06736	Pseudogene	6.27	9.75
AT4G08080	Gypsy-like retrotransposon family (Athila)	6.84	10.74
AT5G32400	Hypothetical protein	6.92	10.32
AT5G32404	Pseudogene	4.90	9.12
AT5G32475	Athila retroelement ORF2-related	5.75	9.37
AT5G32483	Pseudogene	6.41	9.89
AT5G32495	Pseudogene	5.74	9.44
AT5G32517	Pseudogene	5.91	9.34
AT5G33150	Pseudogene	7.33	10.75
AT5G34970	Similar to unknown protein	5.16	8.63

We also applied the above described mSTAD algorithm to the two polyA(±) samples, to detect transcription from unannotated regions. When we subtracted high-confidence segments found in at least one polyA(+) sample from the segments found in both polyA(±) samples, segments totaling less than 100 kb were identified as potential polyA(-) transcripts (Figure 5b). These regions represent less than 0.1% of the entire genome, which appears to be very low compared with results reported for *C. elegans *tiling array studies using the transfrag method [19]. To rule out the possibility that this discrepancy is a computational artifact, we applied the transfrag method to our tiling array data also [46]. This method led to similar estimates of polyA(±) specific transcribed fragments (transfrags), with a combined length of about 250 kb, or 0.2% of the genome (Figure 5b). These results imply that nonpolyadenylated transcripts are much less abundant in *Arabidopsis *than in *C. elegans *and humans [20,47].

### Online resources for visualization of *Arabidopsis *tiling array data

To make our results easily accessible to the research community, we created an online resource that consists of two parts: a web-tool that reports expression values for user-specified genes, and a customized generic genome browser [48].

**Figure 6:**
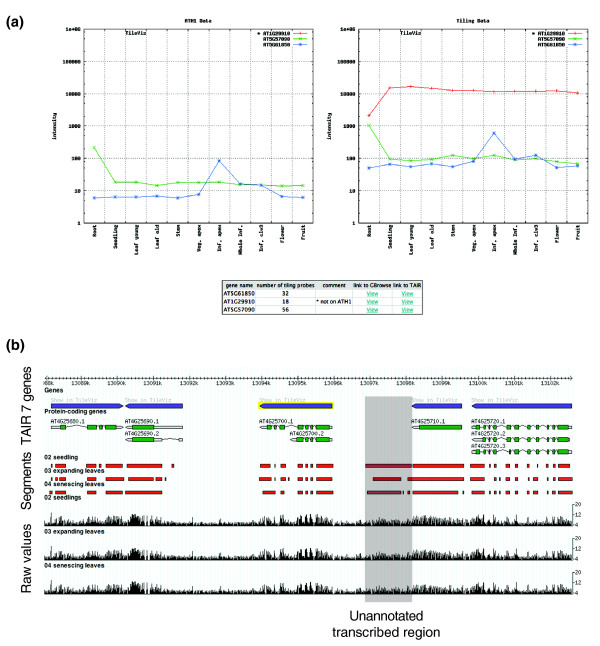
At-TAX online resources for gene expression analysis. **(a) **At-TAX gene expression estimates derived from tiling (right) and ATH1 arrays across all analyzed samples in TileViz. Included in this example is a gene not represented on the ATH1 array (red line). **(b) **Display of predicted expressed segments (middle) and raw hybridization signals (bottom) along the chromosome (top) in a generic genome browser.

The At-TAX gene expression visualization tool can be fed with TAIR (The *Arabidopsis *Information Resource) locus IDs [49]. Expression estimates for input gene(s) are displayed in all analyzed samples and on both ATH1 and tiling arrays, where available (Figure 6a). This not only provides a convenient means of analyzing genes not represented on the ATH1 array, but also allows simple cross-platform comparison. The generic genome browser displays transcriptional active regions as predicted by mSTAD across the genome, as well as all raw expression values for each probe in all analyzed samples [50] (Figure 6b).

## Discussion

In this study, we present an RNA expression atlas, At-TAX, of the *A. thaliana *reference strain Col-0 based on the GeneChip^® ^*Arabidopsis *Tiling 1.0R Array. Expression data have been collected across a series of tissues and developmental stages for the vast majority of annotated genes, including more than 9,000 genes that are not represented on the older ATH1 gene expression array. Moreover, our systematic comparison of the performance of the two arrays should provide valuable information for anybody considering experiments on either one of these two platforms.

### Gene expression profiling with whole genome tiling arrays

Tiling arrays have several advantages compared with focused gene expression arrays such as the ATH1 platform, because tiling arrays allow detection of all transcripts irrespective of their annotation status as well as different splice forms. However, because probes have not been optimized in a similar manner, especially for uniform isothermal hybridization behavior, it has been unclear how broadly suitable they are for routine expression analysis. To address this issue, we used both array types to analyze 11 different samples covering different tissues and developmental stages. The resulting gene expression estimates on both array platforms are highly correlated, including measures of expression changes between tissues. We conclude that whole genome tiling arrays are indeed an appropriate tool for standard gene expression analyses. However, expression estimates derived from the two different platforms can differ for various reasons, indicating that expression data must be interpreted carefully. Discrepancies are often due to the selection of probes on the ATH1 arrays, which are biased towards the 3' end of transcripts and sometimes overlap, thus violating assumptions of independence. Conversely, expression analysis with tiling arrays can be inaccurate for small genes represented by very few probes, especially if these have unfavorable hybridization properties. Uncertainty in gene annotations is another source of error, because expression may erroneously be measured from intronic probes.

Compared with the ATH1 array, a disproportionately high number of genes that are represented only on the tiling array produced very low hybridization signals. This is not unexpected because the genes selected for the ATH1 array were supported by cDNAs and ESTs, whereas the tiling array includes hypothetical genes that lack any experimental evidence of expression. In addition, the number of annotated pseudogenes in *A. thaliana *has been increasing dramatically. The first annotation released in 2001 (TIGR1) contained 1,274 pseudogenes, whereas the recent TAIR7 annotation includes 3,889 pseudogenes [11].

### The dark matter of the *Arabidopsis *genome

Identification of unannotated transcribed regions is a major motivation for tiling array experiments. That our segmentation algorithm generated highly reliable predictions is evident from the observation that there was very good overlap with annotated genes as well as high success rates for RT-PCR validation experiments. Despite extensive cDNA cloning and previous use of tiling arrays (for example, [14]), we could detect more than 1,000 additional transcripts. We found that exonic regions in the different tissues comprise on average about one-third of the genome. Despite the finding of unannotated transcripts, the ratio of annotated exons to polyA(+) transcripts detectable on tiling arrays appears to be much higher in *Arabidposis *than in some other organisms [51]. Interestingly, tiling array analysis of *Arabidopsis *mutants impaired in DNA methylation or RNA quality control has revealed more than 200 noncoding transcripts that are normally transcriptionally silenced, indicating that the *Arabidopsis *genome has at least the potential to generate a large number of transcripts from intergenic regions [31,32].

### The nonpolyadenylated *Arabidopsis *transcriptome

Tiling array studies of human and *C. elegans *indicated that about half of all transcripts are not polyadenylated [20]. In contrast, our data suggest that nonpolyadenylated RNAs make a more limited contribution to the *Arabidopsis *transcriptome. It is already known that specific classes of plants transcripts are generated in a different manner than in animals. For example, some human microRNA precursors are transcribed by RNA polymerase III and hence are not polyadenylated, whereas *Arabidopsis *microRNA precursors feature characteristics of RNA polymerase II generated transcripts [52,53]. Another reason might be differences in 3' end processing. For example, histone mRNAs in land plants are polyadenylated, which is in contrast to histone mRNAs in animals, which are subject to a unique form of 3' end processing resulting in a hairpin that protects the 3' end from RNA degrading enzymes [54-58].

We found that many nonpolyadenylated RNAs in *Arabidopsis *are derived from pseudogenes and transposons. Several examples of actively transcribed pseudogenes have been reported [59], and many pseudogenes become transcriptionally activated in methylation-deficient mutants [31]. Known mechanisms for the transcriptional silencing of pseudogenes involve small RNAs that are generated through the RNA-dependent-RNA-polymerase (RDR)2/DICER-LIKE 3 biogenesis pathway [60,61]. Interestingly, improperly terminated, nonpolyadenylated RNAs derived from transgenes can be subject to a silencing pathway that involves another RNA-dependent-RNA-polymerase, namely RDR6, which can use both polyadenylated and nonpolyadenylated transcripts as a substrate [62,63]. Therefore, our observation that RNAs corresponding to a subset of pseudogenes are much more abundant in the polyA(±) fraction is compatible with a scenario in which these pseudogenes are transcribed into polyA(-) RNAs that subsequently serve as template for RDR-dependent amplification. However, transcripts from some pseudogenes are also detectable in polyA(+) samples. These pseudogenes might either be transcribed into both polyA(+) RNAs and polyA(-) RNA or, alternatively, polyA(-) RNAs derived from polyA(+) RNAs accumulate during RNA amplification and processing steps.

### Outlook

We have demonstrated that the use of the GeneChip^® ^Arabidopsis Tiling 1.0R Array for routine expression analyses does not have any apparent disadvantages compared with the ATH1 array. Rather, it has many advantages, including the ability to provide information on genes that are not represented on ATH1, as well as the ability to analyze additional aspects of gene expression, such as alternative transcript initiation and 3' end formation or splicing, all of which might be under physiological or developmental control [64,65]. Tiling arrays might be the platform of choice to further resolve transcriptional activity over developmental stages and cell types, especially when combined with techniques for the isolation of specific cells by laser microdissection or cell sorting (for review [66]).

## Materials and methods

### Plant material and RNA isolation

Wild-type Col-0 and *clv3*-7 plants [37] were grown on soil or on solid MS medium under continuous light at 23°C. Additional data file 1 describes each sample. Tissue samples were frozen in liquid nitrogen and total RNA was isolated using the RNeasy Plant Mini Kit (Qiagen, Hilden, Germany). RNA integrity was determined on a Bioanalyzer with the RNA 6000 Series II Nano kit (Agilent, Santa Clara, CA, USA).

### Probe preparation and array hybridization

For synthesis of probes (targets) for ATH1 and tiling arrays, 1 μg of total RNA was used as template for generation of cRNA using the MessageAmp II-Biotin Enhanced Kit (Ambion, Austin, TX, USA). We followed the manufacturer's instructions with one exception; for tiling arrays, biotinylated NTPs were replaced by unmodified NTPs (stock solution 25 mmol/l each). Sixteen micrograms of biotinylated cRNA (for ATH1 arrays) was fragmented using 5× Fragmentation Buffer. Seven micrograms of unmodified cRNA (for tiling arrays) was converted into dsDNA (GeneChip^® ^WT Double-Stranded cDNA Synthesis Kit; Affymetrix Inc.) and dsDNA was purified using the MinElute Reaction Cleanup Kit (Qiagen). A total of 7.5 μg dsDNA was fragmented and labeled using the GeneChip^® ^WT Double-Stranded DNA Terminal Labeling Kit (Affymetrix Inc.). Targets were hybridized to ATH1 and *Arabidopsis *Tiling 1.0R arrays for 14 hours at 42°C, washed (Fluidics Station 450, wash protocol EukGE-WS2_V4 for ATH1 arrays or wash protocol FS450_0001 for tiling arrays) and scanned using a GeneChip^® ^Scanner 3000 7 G.

For comparison of polyA(+) and polyA(±), rRNA was depleted from 10 μg total RNA using RiboMinus™ Yeast Transcriptome Isolation Kit (Invitrogen) and an *Arabidopsis *specific RiboMinus™ LNA oligonucleotide mix kindly provided by Invitrogen, Carlsbad, CA, USA. rRNA depleted RNA was precipitated and resuspended in 12 μl water, from which 11 μl were used for reverse transcription using MessageAmp II-Biotin Enhanced Kit (Ambion) with an oligo-dT-T7 primer (MessageAmp II-Biotin Enhanced Kit) or a random-T7 primer (included in the GeneChip^® ^WT Amplified Double-Stranded cDNA Synthesis Kit; Affymetrix Inc.). All subsequent steps were performed exactly as described above.

## Repetitive probe annotation

To assess the potential of each 25 mer oligonucleotide probe on the tiling array to crosshybridize to transcripts from different locations, we determined whether its sequence occurred more than once in the *A. thaliana *genome. To this end we applied a method proposed previously [67], which annotates 25 mers occurring as exact duplicates elsewhere in the genome, those which align with identity at the innermost 21 nucleotides, and those that have a single mismatch in the 25 mer alignment. Probes with exact 25 mer matches were excluded from tiling array expression measurements, and all types of repetitive probes were used to annotate and filter exon segments predicted by mSTAD and transfrags.

### Probe set definitions

In order to analyze annotated genes, we mapped tiling probes to *Arabidopsis *gene models as per TAIR7 annotation [68]. Probe sets for individual genes were defined as follows. From all probes mapped to exons (either coding or untranslated region) in their entire length, we retained only those for expression analysis that correspond to constitutive exons in all annotated splice forms of the same gene. We further excluded probes that mapped to more than one (overlapping) gene model, and in order to reduce cross-hybridization artifacts we also removed repetitive probes whose 25 mer sequence occurred multiple times in the genome. For expression measurements from tiling arrays we only considered the set of 30,228 annotated genes that are represented by at least three probes.

For the ATH1 array, probe sets were defined according to the *A. thaliana *CDF version 10 provided by the Microarray Lab at the Molecular and Behavioral Neurobiology Institute of the University of Michigan [69].

### Expression estimates

In order to minimize artificial expression level differences between platforms only resulting from differences in the computational analyses procedures, the RMA method was applied to hybridization data from both platforms [39]. RMA proceeds in three steps. First, background correction and quantile normalization were applied before gene expression levels were calculated with the median polish method. Data from ATH1 arrays were analyzed using the RMA implementation in the Bioconductor package affy [70-72]. For the analysis of tiling arrays, we constructed a pipeline that combined the same background and quantile normalization methods from Bioconductor (BufferedMatrixMethods package by BM Bolstad), with the median polish routine extracted from Bioconductor sources (preprocessCore package by BM Bolstad) and adopted for the analysis of custom probe sets.

### Detection of differentially expressed genes and CAT plot analysis

We applied the Rank Product method (Bioconductor package RankProduct) [40,73] to identify significantly expressed genes at a cut-off of *P *< 0.05. The *P *values were also used to assess platform concordance by CAT analysis, in which gene lists ordered by *P *value were compared between platforms. The proportion of most significant genes in common between platforms was plotted as a function of list sizes increasing in steps of ten [42]. As a measure of tissue-specific expression, z scores were calculated as described by Schmid and coworkers [5].

### Segmentation of tiling array data

We preprocessed the hybridization signal to reduce a bias due to divergent probe sequences using a transcript normalization method [35,74] and subsequently applied a modified version of the mSTAD algorithm [35].

For each sample, we trained mSTAD separately on mean intensities across replicates and used the trained instance only for prediction of array data from the same sample. To obtain unbiased whole-genome predictions we employed cross-validation. After splitting the genome between pairs of neighboring genes, one instance of mSTAD was trained on 500 of these genic regions and hyper-parameters were tuned on another 500 genic regions. We trained and tuned a second instance of mSTAD on two further disjoint sets of 500 genes each. For region-wise whole-genome predictions, we chose the mSTAD instance that had not seen the particular region during training and hyperparameter tuning (or a random instance if neither of them had). From the predicted labeling of tiling probes we extracted exon segments by assigning the genomic coordinates corresponding to the start of the first and the end of the last probe of a run of consecutive exon labels. The resulting segmentations are available as gff files and visualized in the At-TAX Generic Genome Browser.

Prediction accuracy was determined on genomic regions that had not been used for training or parameter tuning of the mSTAD instance evaluated. Sensitivity and specificity were assessed in comparison to annotated genes on a per-probe level as well as for the overlap between annotated and predicted exons. Figure 4a shows mean performance across 1,000 genic test regions (with at least five probes annotated as exonic and at least ten probes in total) chosen randomly for each of the mSTAD instances used to make whole-genome predictions for root data. Accuracy on probe level was also calculated for whole-genome (test) predictions for all other samples (see Additional data file 2).

To determine overlap with annotated regions, we used the TAIR7 annotation [11] and direct alignments with EST and cDNA sequences (downloaded from TAIR on 15 August 2007) [75]. Sample-specific segments were obtained as residual after computing the overlap between predicted exon segments in the tissue of interest to those from all other tissues (Figure 4d). Similarly, we obtained predictions specifically made for polyA(±) conditions as exon segments that were predicted for both polyA(±) samples (ones that overlapped between samples), but did not overlap to predictions for any polyA(+) sample (Figure 5c).

### RT-PCR validation

One microgram of RNA from seedlings and young leaves was treated with DNaseI (MBI Fermentas, St. Leon-Rot, Germany) and converted into cDNA using the RevertAid™ First Strand cDNA Synthesis Kit (MBI Fermentas). One microliter of the resulting cDNA solution was used as a template in a PCR reaction with primers lying within the predicted transcribed region. The sizes of PCR products ranged from about 150 to 300 bp. A complete list of all used primers is available in Additional data file 3.

### Computation of transcribed fragments (transfrags)

As an independent method to compare transcriptional activity between polyA(+) and polyA(±) samples, we computed transfrags as described previously [76] and implemented in the Affymetrix Tiling Analysis Software version 1.1 build 2. In order to select optimal parameters, we evaluated transfrags generated for root tissues for 900 different combinations of parameters in comparison with annotated genes (bandwidth in steps of 25 between 50 and 150, signal threshold between 5 and 13, minimum run in steps of 20 between 20 and 100, and maximum gap in steps of 20 between 40 and 100). As optimal setting for all transfrag computations we chose the one with maximal sensitivity at a precision similar to mSTAD predictions (bandwidth 100, signal threshold 6, minimum run 100, maximum gap 40; see Additional data file 8). Among nonrepetitive transfrags (at most 25% repetitive probes) comprising at least four probes and without overlap to annotated transcripts, the ones specific to polyA(+) or polyA(±) samples were computed the same way as for high-confidence mSTAD predictions (Figure 5d).

## Abbreviations

At-TAX, Arabidopsis thaliana Tiling Array Express; bp, base pair; CAT, correspondence at the top; dsDNA, double-stranded DNA; EST, expressed sequence tag; kb, kilobases; LNA, locked nucleic acid; mSTAD, margin-based segmentation of tiling array data; PCC, Pearson correlation coefficient; RDR, RNA-dependent-RNA-polymerase; RMA, robust multichip analysis; RT-PCR, reverse transcription polymerase chain reaction; TAIR, The Arabidopsis Information Resource.

## Authors' contributions

SL, GZ, MV, BS, GR, and DW designed the study. SL carried out target preparation and array hybridization. GZ, SRH, TS and NN developed tools for tiling array analysis. GZ, SRH, SL, and TS analyzed the data. TS and CKW developed online visualization tools. SL, GZ, GR, and DW wrote the manuscript. All authors read and approved the final manuscript.

## Additional data files

The following additional data are available with the online version of this paper. Additional data file 1 lists all analyzed samples, including growth conditions and plant age. Additional data file 2 shows segmentation accuracy of mSTAD. Additional data file 3 lists oligonucleotide primers that were used for RT-PCR validation of new transcripts. Additional data file 4 shows correlation between platform concordances and probe numbers on the ATH1 array. Additional data file 5 shows segmentation accuracy achieved by mSTAD along the five *Arabidopsis *chromosomes. Additional data file 6 shows the results of all RT-PCR validation experiments. Additional data file 7 shows a comparison of mean hybridization intensities in random-primed and oligo-dT-primed samples. Additional data file 8 shows a comparison of segmentation accuracy for mSTAD and the transfrag method. Additional data file 9 contains gene identifiers with corresponding expression values and z-scores in all samples we analyzed.

## Supplementary Material

Additional data file 1 Listed are all analyzed samples, including growth conditions and plant age. Click here for file

Additional data file 2 Shown is the segmentation accuracy of mSTAD. Click here for file

Additional data file 3 Shown are the oligonucleotide primers that were used for RT-PCR validation of new transcripts. Click here for file

Additional data file 4 Shown is the correlation between platform concordances and probe numbers on the ATH1 array. Click here for file

Additional data file 5 Shown is the segmentation accuracy achieved by mSTAD along the five *Arabidopsis *chromosomes. Click here for file

Additional data file 6 Presented are the results of all RT-PCR validation experiments. Click here for file

Additional data file 7 Presented is a comparison of mean hybridization intensities in random-primed and oligo-dT-primed samples. Click here for file

Additional data file 8 Presented is a comparison of segmentation accuracy for mSTAD and the transfrag method. Click here for file

Additional data file 9 Presented are gene identifiers with corresponding expression values and z-scores in all samples we analyzed. Click here for file
