# Supplementary material for: At-TAX: a whole genome tiling array resource for developmental expression analysis and transcript identification in Arabidopsis thaliana
Source: Genome Biol. 2008 Jul 9;9(7):R112. doi: 10.1186/gb-2008-9-7-r112 (PMC2530869; doi:10.1186/gb-2008-9-7-r112)
Supplement: Additional data file 1 — Listed are all analyzed samples, including growth conditions and plant age. [file gb-2008-9-7-r112-S1.doc]

**Table S1.** Analyzed samples.

| **Sample** | **Genotype** | **Description** |
| --- | --- | --- |
| 1 | WT (Col) | Roots, 7 days |
| 2 | WT (Col) | Seedlings, aerial parts, 7 days |
| 3 | WT (Col) | Expanding leaves, #4 10 days |
| 4 | WT (Col) | Senescing leaves, 35 days |
| 5 | WT (Col) | Stem, 2nd internode |
| 6 | WT (Col) | Vegetative shoot meristem, 7 days |
| 7 | WT (Col) | Inflorescence shoot meristem, 21 days |
| 8 | WT (Col) | Whole inflorescences to floral stage 9, 21 days |
| 9 | *clv3-*7 (Col) | Whole inflorescences (*clv3-*7), 21 days |
| 10 | WT (Col) | Flowers, stage 15, 21+ days |
| 11 | WT (Col) | Fruits, carpels stage 15, 21+ days |

Plants were grown in continuous light at 23°C.
