# Supplementary material for: At-TAX: a whole genome tiling array resource for developmental expression analysis and transcript identification in Arabidopsis thaliana
Source: Genome Biol. 2008 Jul 9;9(7):R112. doi: 10.1186/gb-2008-9-7-r112 (PMC2530869; doi:10.1186/gb-2008-9-7-r112)
Supplement: Additional data file 2 — Shown is the segmentation accuracy of mSTAD. [file gb-2008-9-7-r112-S2.doc]

**Table S2.** Segmentation accuracy of mSTAD by sample, instance and chromosome.

|  | **Accuracy on probe level (percent)** | | | | | | |
| --- | --- | --- | --- | --- | --- | --- | --- |
| Sample | mSTAD instance 1 | mSTAD instance 2 | Chr 1 | Chr 2 | Chr 3 | Chr 4 | Chr 5 |
| 1 | 81.0 | 79.5 | 81.3 | 79.3 | 79.2 | 79.7 | 80.9 |
| 2 | 80.4 | 80.2 | 81.3 | 79.1 | 79.6 | 79.8 | 81.0 |
| 3 | 78.5 | 79.3 | 79.8 | 78.0 | 78.1 | 78.6 | 79.5 |
| 4 | 78.6 | 80.1 | 80.2 | 78.3 | 78.6 | 79.1 | 80.0 |
| 5 | 78.6 | 79.4 | 79.8 | 78.3 | 78.2 | 78.5 | 79.8 |
| 6 | 78.0 | 76.9 | 78.2 | 76.5 | 76.7 | 77.0 | 78.1 |
| 7 | 78.5 | 78.2 | 79.1 | 77.7 | 77.6 | 77.7 | 79.0 |
| 8 | 76.8 | 77.0 | 77.7 | 76.4 | 76.2 | 76.4 | 77.5 |
| 9 | 79.3 | 80.3 | 80.6 | 79.0 | 79.0 | 79.4 | 80.5 |
| 10 | 78.2 | 78.3 | 79.0 | 77.4 | 77.5 | 77.8 | 78.9 |
| 11 | 78.1 | 77.2 | 78.4 | 77.1 | 76.9 | 77.1 | 78.2 |
| 12 | 81.7 | 82.2 | 82.8 | 80.9 | 81.0 | 81.5 | 82.8 |
| 13 | 80.9 | 82.6 | 82.7 | 80.7 | 81.0 | 81.4 | 82.5 |
| 14 | 78.0 | 78.4 | 79.0 | 77.5 | 77.5 | 77.8 | 78.9 |
| 15 | 77.6 | 78.1 | 78.7 | 77.0 | 77.1 | 77.5 | 78.3 |
