# Supplementary material for: At-TAX: a whole genome tiling array resource for developmental expression analysis and transcript identification in Arabidopsis thaliana
Source: Genome Biol. 2008 Jul 9;9(7):R112. doi: 10.1186/gb-2008-9-7-r112 (PMC2530869; doi:10.1186/gb-2008-9-7-r112)
Supplement: Additional data file 3 — Shown are the oligonucleotide primers that were used for RT-PCR validation of new transcripts. [file gb-2008-9-7-r112-S3.doc]

**Table S3. Oligonucleotide primers used for RT-PCR validation of new transcripts.**

| **Segment #** | **Chr** | **Start** | **Stop** | **Primer sequence (forward)** | **Primer sequence (reverse)** |
| --- | --- | --- | --- | --- | --- |
| 1 | CHR4 | 15503948 | 15504280 | GAT ACT CGC ACA ACA ATC ATG | CAT TAT TTC TCC TCT CTA TTG |
| 2 | CHR5 | 20173286 | 20173594 | CTG TCT GCC TTT TTC ACA TAG | TCT CTC TCT TCT TAT CTT TAG |
| 3 | CHR2 | 8002070 | 8002315 | CAT CAA TCA TAT CTT CAA CAG | CAC ACT GAA CTC TGG AAG ATG |
| 4 | CHR2 | 14914411 | 14914651 | ACA CAT CTC GAT ATC TAA GC | CGC TTA GTG CTC CGG TAA AAC |
| 5 | CHR5 | 18776016 | 18776150 | AAA GCT CAA CAA CTT AAT AAG | ATG TGG AGC ATC TTC ATT ATG |
| 6 | CHR4 | 13097119 | 13097927 | GTT TGA TTC TCT TGC GCT TTG | GGA TCA TGA TCA GAG TTT CTG |
| 7 | CHR5 | 2396793 | 2397150 | TCA CCA AGA TAA CCC CAA ATG | TCA CTA GTT GGC ACA GCC ATG |
| 8 | CHR3 | 20831252 | 20831453 | TTA TTC CAA ATC TAA GGA AAG | CCG TTA GGA GGA GAA GTG AAG |
| 9 | CHR4 | 9167909 | 9168108 | GAC ATC AGG AGC TAT GAC ATG | AAC TGA AGA GGG CAG TGT TAG |
| 10 | CHR3 | 3607886 | 3608056 | TAC CAG AAA CTG AGC TCC TAG | CAT CTT TCT CAA CCG CCG TAG |
| 11 | CHR1 | 1282198 | 1282332 | ATT AGG ACA ATG CCT CTG GTG | TTC AAT GAC TTT GCT GCA GAG |
| 12 | CHR1 | 23284145 | 23285271 | AGC CAA CCT CTC GGA ACC AAG | GTG GTT AGG CTT CTG TCC TTG |
| 13 | CHR3 | 872928 | 873565 | GCT AGA GAC TGA GTA ATG ATG | CAC ACA TCT TAC AAG CAT TTG |
| 14 | CHR4 | 7846881 | 7847467 | ATC TGA CAA CTG GAA TAA AGT | AGG TAC GAT ACT CTA GTC TAG |
| 15 | CHR1 | 11077435 | 11077975 | AGC GTA TAC GTA CCA GAG ATG | ACA ATA ATG GCT CAA GTG GTG |
| 16 | CHR1 | 2246810 | 2247304 | CAA TAA AAA GGA GGG ATC AAG | TGT TCT GTT CTC AAT GGA ATG |
| 17 | CHR2 | 18331818 | 18332320 | CAA AAA CAA CAC AAT GGG AAG | TTT GCA TGA GTA TGA AGG AAG |
| 18 | CHR4 | 13318797 | 13319255 | AGA ACT ACA TTA AGA ACA GAG | TAT CAG CAT CAC TTT CGT CTG |
| 19 | CHR5 | 19649991 | 19650454 | ATC CGT TTC TAC AAG ACT CAG | AGT GTT GGT ATA ACA CAG TAG |
| 20 | CHR1 | 8919031 | 8919446 | AGG AAG AGA CAA GTA CAG GAG | TCC ACG TCC CAA CAT GAA G |
| 21 | CHR1 | 23405212 | 23405627 | AGA ACG ACC TCG AAG TTG CTG | TGT TTC AGT AAT CTC CTA GTG |
| 22 | CHR2 | 14909684 | 14910095 | CAA GAA TCC AAC CCA AAC AAG | TCG CCA TGA TGG AGT ATC GAG |
| 23 | CHR3 | 5570652 | 5571067 | ATG ATC TAT CTG AAG AAT CAG | TCC ACG ATT TCT ATG GCT TCG |
| 24 | CHR1 | 23036401 | 23036700 | TGT ATG ACG AAA CTA ACG CTG | TCC AAT AGT TGA AGA CTG CTG |
| 25 | CHR1 | 25578140 | 25578482 | TAC ATT TGG GCA GCT GTT GTG | AAG AAG CAG AAT ACA TTC ATT G |
| 26 | CHR3 | 1397141 | 1397446 | ATA GAT TTC TCT GAT TTG CAG | AAA TTT CTC AGT CTC GCC GTG |
| 27 | CHR3 | 8226184 | 8226524 | GCA GAT ATA TTA GTT CAG CAG | CAG TAC TCT AAC AAT TCT TAT G |
| 28 | CHR1 | 9070774 | 9071028 | CAA CAA GGA AAA TCA CTG AAG | GAG ACT ATT GTC GTT TGG TAG |
| 29 | CHR1 | 12087115 | 12087358 | CTC GAG AGT TCT TGA CTT CTG | TGA CTA AAA TAG AAC GTC CAG |
| 30 | CHR1 | 11675562 | 11675776 | TTC TTC CTC AAG CTA CCT ATG | AGG AAA CGA GGG TAT GGA CTG |
| 31 | CHR1 | 28372225 | 28372424 | CCT ATG TGA TCC AGT GAT CAG | AAT GTG TCA ACC CGA TAT CAG |
| 32 | CHR4 | 5564403 | 5564571 | TTT GCG GTG GTG GAA GAA CTG | TCC TCG CTG TGA TCG AAT AAG |
| 33 | CHR1 | 4680125 | 4680262 | AGA GCC GTC CGA TCA ACA CAG | GGT CTA AGT TGC CAC ATC ATC |
| 34 | CHR1 | 16418537 | 16419850 | AGC TGA GAC AAA CAA ATG TAG | GAG CAA TGA CGC CGT CAT GAG |
| 35 | CHR2 | 8796734 | 8797389 | TAG GGA TGT AGA CAT AGC GAG | AAT CAA GGT CAT CTC CTT CTG |
| 36 | CHR3 | 15213 | 15743 | GCA TCA GAG AAT GGT ATG GAG | ATG ACA TGG GTA TCA CTG TTG |
| 37 | CHR4 | 16200564 | 16201009 | ACC CTT GAG AAA TAC ACA CAG | TAT CGT TAC GCC ATG ATC GAG |
| 38 | CHR5 | 16174040 | 16174473 | GCG TAG ACA CCA TCG CAC TG | ACC TTT AGA CCA CGA CTG CAG |
| 39 | CHR5 | 16728686 | 16729099 | ACA ACA GAA GTC ATC AAG ATG | CAA GCC GTA CTC TCT CAG AAG |
| 40 | CHR3 | 4699627 | 4700590 | GCC TAG AAG CCA TAG CAG CAG | CAG AAG AAT CTG AGG CAT CAG |
| 41 | CHR5 | 26614538 | 26615492 | ATT GAG AGA ATG ATG GCT ATG | ATC AGA TAT CGA ACC GCA GAG |
| 42 | CHR5 | 1042453 | 1043112 | AAG ATA CAT GTA TGT ATG TAG | GAA CTA CAC TAG TCA CGC ATT G |
| 43 | CHR3 | 16061040 | 16061612 | GGA ACT TGT CAG AAA GGA GAG | ACA TAT CAT AAA GCA AGC CAG |
| 44 | CHR4 | 13213949 | 13214281 | AAA TGT ATT GAT CCG TAG GTG | TGG TTG CAG ACA TTA CCT CAG |
| 45 | CHR5 | 4012328 | 4012603 | AAG TGG TTG TGA TTG AGA ATG | TTC GTC CAC GAC AAC GTC CAG |
| 46 | CHR5 | 9399081 | 9399325 | AAG AGA GAA GAG AGA TAC CTG | ACT TTT TGA TTC TAC GAG TCG |
| 47 | CHR1 | 11310580 | 11310780 | ACT TCT GGA GAC ATA AGC CAG | AAT ATC CCA TTC TCA AGT GAG |
