# Supplementary material for: At-TAX: a whole genome tiling array resource for developmental expression analysis and transcript identification in Arabidopsis thaliana
Source: Genome Biol. 2008 Jul 9;9(7):R112. doi: 10.1186/gb-2008-9-7-r112 (PMC2530869; doi:10.1186/gb-2008-9-7-r112)
Supplement: Additional data file 4 — Shown is the correlation between platform concordances and probe numbers on the ATH1 array. [file gb-2008-9-7-r112-S4.doc]

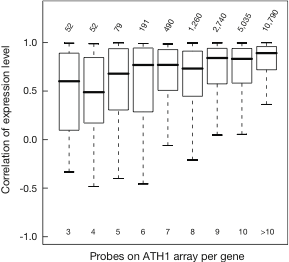


**Figure S1.** Platform concordance and probe number on ATH1 arrays.

Box plots showing expression correlation across platform.
