# Supplementary figures and images for: At-TAX: a whole genome tiling array resource for developmental expression analysis and transcript identification in Arabidopsis thaliana
Source: Genome Biol. 2008 Jul 9;9(7):R112. doi: 10.1186/gb-2008-9-7-r112 (PMC2530869; doi:10.1186/gb-2008-9-7-r112)

**
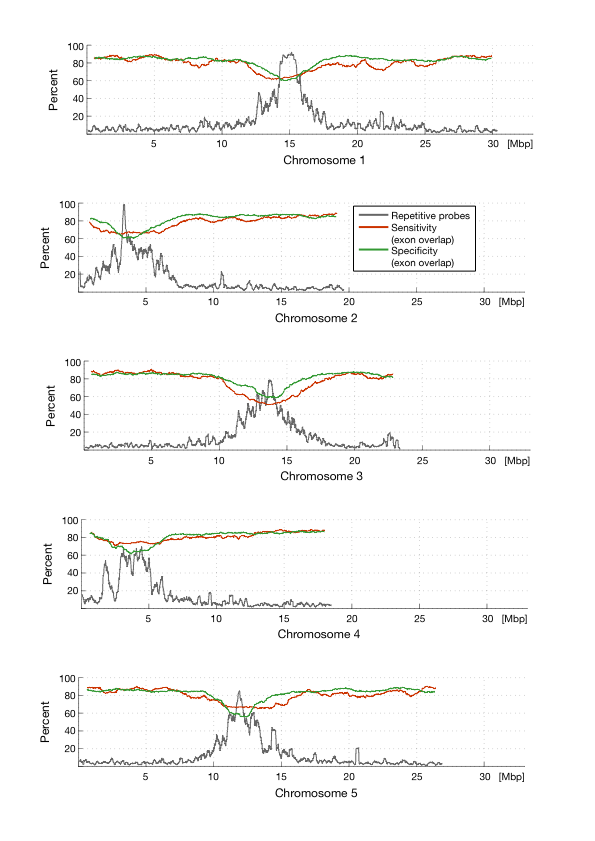
**

**Figure S2.** Segmentation accuracy achieved by mSTAD along the *Arabidopsis* genome.

Supplement: Additional data file 5 — Shown is the segmentation accuracy achieved by mSTAD along the five Arabidopsis chromosomes. [file gb-2008-9-7-r112-S5.doc]
