# Supplementary material for: At-TAX: a whole genome tiling array resource for developmental expression analysis and transcript identification in Arabidopsis thaliana
Source: Genome Biol. 2008 Jul 9;9(7):R112. doi: 10.1186/gb-2008-9-7-r112 (PMC2530869; doi:10.1186/gb-2008-9-7-r112)
Supplement: Additional data file 6 — Presented are the results of all RT-PCR validation experiments. [file gb-2008-9-7-r112-S6.doc]

**
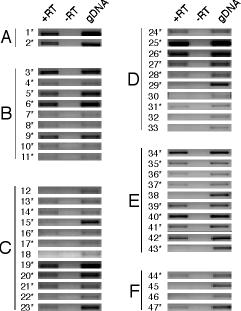
**

**Figure S3.** RT-PCR validation results of novel segments.

Predicted segments were subdivided into **(A)** long segments, high expression level; **(B)** short segments, high expression level; **(C)** long segments, medium expression level; **(D)** short segments, medium expression level; **(E)** long segments, low expression level; and **(F)** short segments, low expression level. Asterisks indicate successful amplification.
