# Supplementary material for: At-TAX: a whole genome tiling array resource for developmental expression analysis and transcript identification in Arabidopsis thaliana
Source: Genome Biol. 2008 Jul 9;9(7):R112. doi: 10.1186/gb-2008-9-7-r112 (PMC2530869; doi:10.1186/gb-2008-9-7-r112)
Supplement: Additional data file 7 — Presented is a comparison of mean hybridization intensities in random-primed and oligo-dT-primed samples. [file gb-2008-9-7-r112-S7.doc]

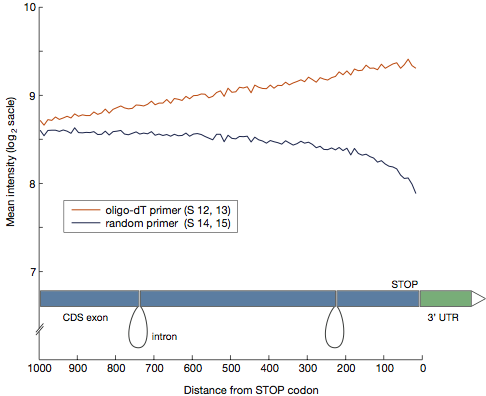


**Figure S4.** Mean hybridization intensity as a function of distance from the 3’ end of coding sequence. UTRs were not considered due to uncertainties in their annotation. Starting from the STOP codon, we averaged hybridization intensities over up to 1 kb of transcribed sequence (excluding introns) of all genes annotated in TAIR7. An example gene is depicted below mean intensities.
