# Supplementary material for: At-TAX: a whole genome tiling array resource for developmental expression analysis and transcript identification in Arabidopsis thaliana
Source: Genome Biol. 2008 Jul 9;9(7):R112. doi: 10.1186/gb-2008-9-7-r112 (PMC2530869; doi:10.1186/gb-2008-9-7-r112)
Supplement: Additional data file 8 — Presented is a comparison of segmentation accuracy for mSTAD and the transfrag method. [file gb-2008-9-7-r112-S8.doc]

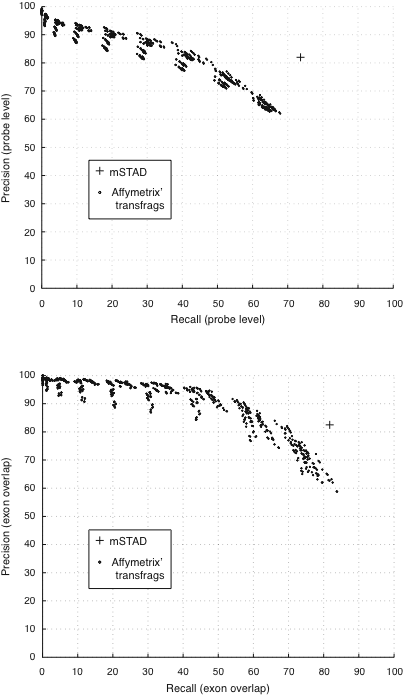


**Figure S5.** Precision-recall curves.

Evaluation of segmentation accuracy for mSTAD as well as various transfrag settings against annotated genes is shown (see inset).
